# Microglia and Beyond: Innate Immune Cells As Regulators of Brain Development and Behavioral Function

**DOI:** 10.3389/fimmu.2018.00698

**Published:** 2018-04-13

**Authors:** Kathryn M. Lenz, Lars H. Nelson

**Affiliations:** ^1^Department of Psychology, The Ohio State University, Columbus, OH, United States; ^2^Department of Neuroscience, The Ohio State University, Columbus, OH, United States; ^3^Institute for Behavioral Medicine Research, The Ohio State University, Columbus, OH, United States; ^4^Neuroscience Graduate Program, The Ohio State University, Columbus, OH, United States

**Keywords:** microglia, brain development, sex differences, synaptic pruning, neurodevelopmental disorders, early life stress, inflammation, behavior

## Abstract

Innate immune cells play a well-documented role in the etiology and disease course of many brain-based conditions, including multiple sclerosis, Alzheimer’s disease, traumatic brain and spinal cord injury, and brain cancers. In contrast, it is only recently becoming clear that innate immune cells, primarily brain resident macrophages called microglia, are also key regulators of brain development. This review summarizes the current state of knowledge regarding microglia in brain development, with particular emphasis on how microglia during development are distinct from microglia later in life. We also summarize the effects of early life perturbations on microglia function in the developing brain, the role that biological sex plays in microglia function, and the potential role that microglia may play in developmental brain disorders. Finally, given how new the field of developmental neuroimmunology is, we highlight what has yet to be learned about how innate immune cells shape the development of brain and behavior.

## Purpose of This Review

Microglia, the brain’s primary resident immune cells, were named and first studied by Pio del Rio Hortega in the 1920s. Since that time, the role of immune cells in the brain and behavior following injury, illness, or infection has been well appreciated. Innate immune cells clearly play a role in the etiology and disease course of multiple sclerosis, Alzheimer’s disease, traumatic brain and spinal cord injury, and brain cancers. Thus, the potential is high that modulating neuroimmune signaling and function will be a viable target for therapeutic interventions. However, innate immune cells in the brain do more than respond to injury and pathological conditions. The last decade has seen an exponential growth in interest in immune cells as regulators of normal and abnormal brain development in response to early life perturbations. In this review, we pursue two main goals. First, we summarize what is currently known about microglia during normal brain development as well as in response to early life stress, infection, and other early life exposures. We focus particularly on areas in which developmental microglia function may be misunderstood, given the much larger and sometimes contrasting knowledge-base on microglia function in the context of injury or neurodegeneration. Second, in each section of the review, we highlight areas of future interest, including where more is unknown than known, where this young field is headed, and where the field may need to refine and complicate the traditional, canonical and potentially dogmatic views of how microglia shape brain function.

## Overview of Microglial Development

**Figure 1:**
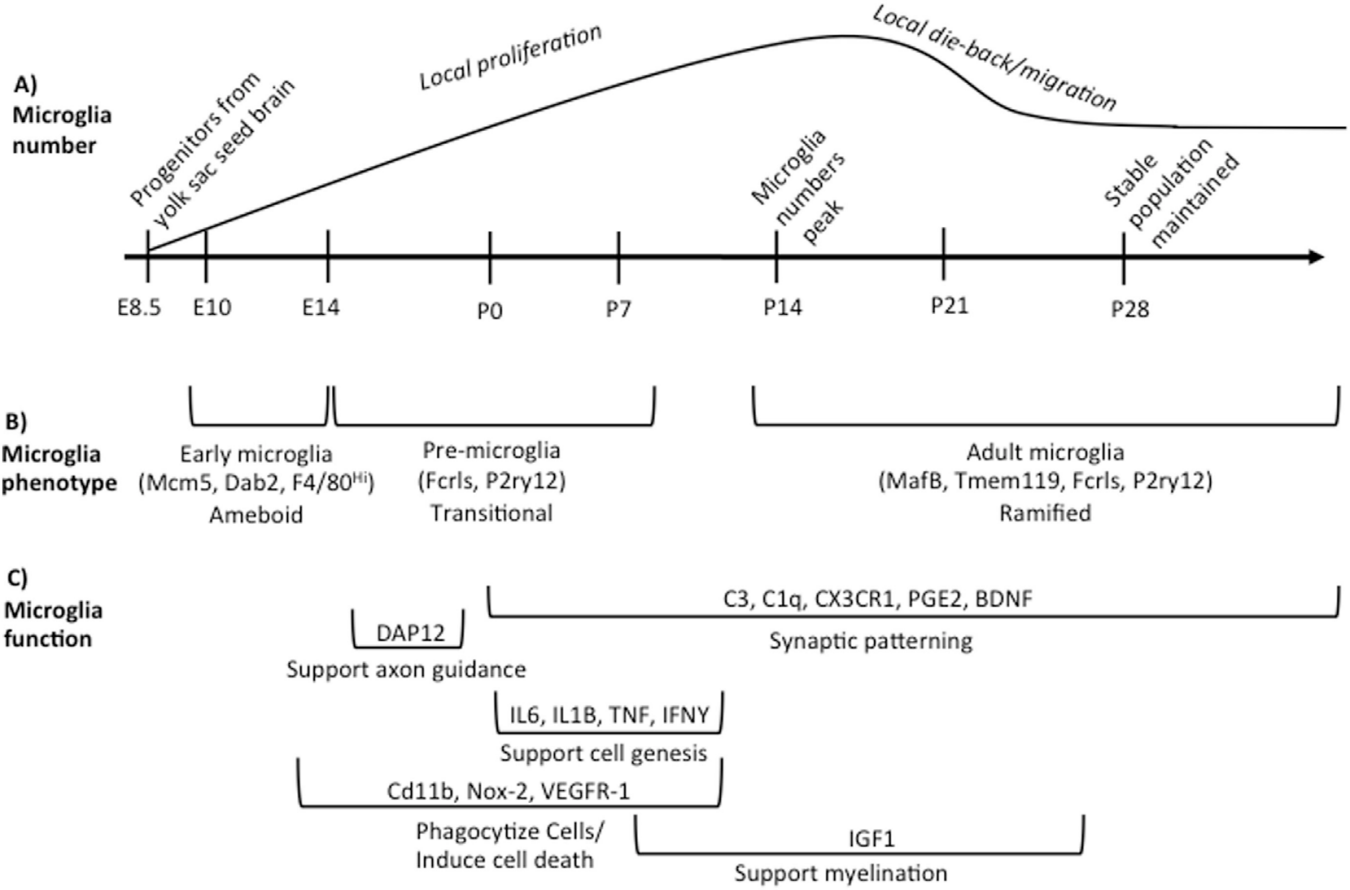
Overview of microglia during brain development. **(A)** illustrates the developmental time points when microglia colonize and proliferate in the developing brain, beginning on embryonic day (E) 8.5. Microglia numbers peak in the rodent brain at postnatal day (P) 14 following local proliferation. Microglia then either die back or migrate from region to region until adult numbers are reached by P28. **(B)** indicates the different phenotypes of microglia across development based on unique gene expression patterns, listed in parentheses, and somewhat distinct morphologies. The brackets refer back to the developmental timeline depicted above in **(A)**. **(C)** summarizes our current state of knowledge regarding which processes of development microglia have been shown to regulate, as well as the molecular factors involved. The brackets refer back to the developmental timeline depicted above in **(A)**, illustrating when research to date has shown that microglia are involved in a given process. These data do not preclude the (likely) possibility that microglia regulate each developmental process beyond the time window indicated, but represent our current state of knowledge. Table 1 lists the publications that were used to design **(C)**.

Microglia are the primary innate immune cells of the brain. They colonize the brain early in brain development. The mechanisms driving microglia colonization and differentiation have only recently been described. In humans, primitive microglia/macrophages are seen near the mesenchymal tissue capillaries before their appearance in neural tissue around 4.5 weeks of gestation and are present in the neural tissue by 5.5 weeks of gestation (1, 2). In rodents, a subset of CD45^-^c-kit^+^ erythromyeloid precursors from the yolk sac use blood circulation to travel to and colonize the mesenchyme surrounding the neural tube beginning at embryonic day (E) 8 (3, 4). In the mesenchyme, the progenitor microglia begin expressing fractalkine receptor (e.g., CX_3_CR1) and downregulate c-kit starting around E9.5 (4, 5). The CX_3_CR1 + microglia progenitors invade the neuroectoderm likely using matrix metalloproteinases, at which point the contribution of peripheral progenitors slows or stops (4, 5). Microglia precursor formation initially depends on cell survival factor (CSF) 1-receptor (CSF1-R) signaling and the transcription factors, PU.1 (SPl1) and interferon regulatory factor 8 (IRF8) (4–6). Interleukin (IL)-34 is likely the predominant CSF1-R ligand during development, as CSF-1 knockout mice do not lack microglia and IL-34 is expressed at greater levels during early brain development (7–9). Microglia colonization can be influenced by fibronectin, macrophage migration inhibitory factor (MIF), fractalkine (e.g., CX3CL1), and CXCL12 (10–13). Once colonization has occurred, microglia locally proliferate in the brain until the second week of postnatal life in rodents. After this peak in numbers, microglia number decreases to adult levels (14–16). This developmental trajectory is summarized in Figure 1.

Microglia are phenotypically and developmentally distinct from peripheral macrophages and other tissue-resident macrophage populations, such as Kupffer cells and aveolar macrophages (14, 17–21). Microglia arise from yolk-sac fetal macrophages, whereas other tissue macrophages arise from precursors generated slightly later in development (20, 21). Hematopoietic cells do not contribute to microglia homeostasis during normal development and adulthood (5). However, peripheral hematopoietic cells may contribute to the microglia pool in the brain in pathological circumstances. For example, following chronic stress and irradiation of the brain that compromise the blood brain barrier, peripheral hematopoietic cells can invade the neural tissue and become part of the microglia/macrophage pool in the parenchyma (22, 23). Together, these data suggest that microglia have a unique developmental origin and tissue environment that drives their specialized development.

## Developmental Microglia are Distinct

Microglia have unique gene expression profiles during different phases of development. Matcovitch-Natan et al. (24) found that microglia express distinct sets of genes that can divide microglia into three distinct groups: early (E10.4-14), pre-microglia [E14-postnatal day (P) 9], and adult microglia (P28 and on). Bennett et al. (14) also found similar developmental changes in microglial gene expression. Some canonical microglia genes are expressed very early in microglia development (e.g., Fcrls, P2ry12) whereas others are only expressed in adult microglia (e.g., MafB, Tmem119) (14, 24). Microglia in the prenatal and early postnatal brain have distinctly different morphologies than microglia in the adult brain. They are largely non-ramified, instead possessing an ameboid morphology until the early postnatal period (25). Bennett et al. (14) also found that microglia begin to adopt a mature phenotype around the end of second postnatal week of life similar to Matcovitch-Natan et al. (24) as indicated by the expression of Tmem119 and a predominately ramified morphology. These developmental differences in microglia gene expression are summarized in Figure 1.

The ameboid morphology seen in developing microglia led to the natural conclusion that microglia were in a constitutively “activated” state in the developing brain, possibly behaving very differently than adult microglia. Recent research has shown that developing microglia do behave differently than adult microglia, but are not “activated” in the same way adult microglia are in response to inflammation or neurodegeneration. For example, there is not substantial gene expression overlap between lipopolysaccharide (LPS)-stimulated microglia from the adult brain and microglia from a control neonatal brain (14). Additionally, a unique Cd11c + microglia population exists in the developing white matter areas; yet, these Cd11c + microglia are not similar to Cd11c + microglia present in a rodent model of multiple sclerosis (experimental autoimmune encephalomyelitis) in terms of their gene expression profile (26). In contrast, microglia isolated from different neurodegenerative disorders share a gene expression profile (high Trem2, ApoE, and Cd11c) (27), which underscores that developmental microglia are phenotypically distinct from “activated” microglia seen in neuropathological conditions. Microglia do express some markers during development that are commonly associated with “activation” or neurodegeneration such as CD11c and CD68, but together the aforementioned data show that developmental microglia are not interchangeable with adult “activated” microglia. Indeed, notions of “activated” or “quiescent” microglia are certainly too simplistic for the dynamic and busy cells during the developmental period. Instead, the field has quickly moved beyond such nomenclature in favor of assessing microglia gene expression, phagocytic capacity, altered density, and/or ultrastructure [for example, see Ref. (24, 28–30), respectively]. Developmental microglia are clearly unique and easily distinguishable from adult “activated” microglia, thus analysis of microglia by morphology alone is likely to be uninformative, or at its best, an imperfect proxy measure of their gene expression profile or function. Other recent studies also suggest that macro-environmental challenges induce complex changes in microglial gene expression during development that differ from those changes seen later in life in response to perturbations (see The Macro-Environment Drives Microglia Function), again underscoring that developing microglia are unique.

## Diverse Microglia Functions in Development

Microglia regulate brain development primarily through two routes: the release of diffusible factors and phagocytosis. Microglia phagocytize many products in the brain, including synaptic elements, living cells, dying or dead cells, and axons. Microglia also support myelination/oligodendrogenesis, neurogenesis, axon fasciculation, induce cell death or cell survival, and stimulate synaptic formation and maturation *via* the release of diffusible factors (11, 31–40). Many factors contribute to microglia phagocytosis of cells, such as vascular endothelial growth factor (VEGF), NADPH oxidase (Nox) 2, and Tyrobp/DAP12 either through recognition of cells marked for removal or by inducing cell death (40, 41). Microglia phagocytosis of neural progenitors increases toward the end of the developmental neurogenesis period in rodents and primates; however, it is unknown if this increase is instigated by microglia or if progenitor cells begin to express a tag that recruits microglia (31). There are several “tags” that regulate cellular phagocytosis such as phosphatidyl serine, complement, calreticulin, ATP, and sialic acid (42, 43). Receptors on microglia that bind to these “tags” include MERTK, vitronectin, CR3, siglecs, and SIRP1α. Microglia also continue to shape the rate of cell genesis throughout life in the hippocampus *via* phagocytosis (44).

Microglia also phagocytize synapses, which has been most elegantly demonstrated in the dorsolateral geniculate nucleus of the thalamus (dLGN). During the early postnatal period, microglia phagocytize “weak” retinal ganglion synapses in the dLGN by recognizing complement component 3 (C3) through the complement receptor 3 (CR3) (36, 45, 46). Decreased retinal ganglion activity and/or increased transforming growth factor (TGF) β signaling in retinal ganglion cells stimulates the synthesis of complement component 1q (C1q) by retinal ganglion cells, which potentially initiates the complement cascade (36, 45, 46). The complement system is not the only signaling system that mediates phagocytosis. Recent work has identified interleukin-33 as an astroglial-secreted factor that regulates microglia synaptic phagocytosis through the interleukin 1 receptor-like 1 (IL1RL1) in the developing thalamus and spinal cord (47).

In the dLGN, there are two periods of intense microglial synaptic phagocytosis. The first period is during the early neonatal period after initial synapses are formed, and the second period is during the juvenile when the fine connections are pruned (48). Microglia also prune synapses in the hippocampus during the second week of life (12). Other factors that regulate synaptic pruning include Class I major histocompatibility complex genes (H2-K^b^ and H2-D^b^) and pentraxins (49, 50); however, it is unknown if microglia are directly involved in these synaptic pruning mechanisms. H2-K^b^ and H2-D^b^ colocalize with C1q, suggesting that microglia may be involved (50). Additionally, microglial synaptic pruning has only been closely investigated in the developing dLGN and hippocampus; thus, it is unknown whether or how microglia contribute synaptic pruning in other brain areas.

Microglia support cell genesis and/or cell health through the synthesis and release of insulin-like growth factor 1(IGF1) and a variety of cytokines that include tumor necrosis factor (TNF) α, IL 1β, IL6, and interferon (IFN) γ (26, 32, 37). Microglia can also stimulate dendritic spine and synapse formation *via* the release of brain-derived neurotrophic factor (BDNF), prostaglandin E2 (PGE2), and IL-10 (33, 51, 52). The major mechanisms and molecules through which microglia have been shown thus far to regulate brain development are summarized in Figure 1 and Table 1.

**Table 1:** Summary of the known major developmental functions of microglia, the ages at which they have been observed, the brain regions in which they have been observed, the major impact of the finding, and the reference associated with the findings. These citations are the basis for the schematic in Figure 1.

Developmental function	Age	Brain area	Notable findings	Reference
Synaptic patterning	P12–15	Hippocampus	CX3CR1, social behavior	Paolicelli et al. (12) and Zhan et al. (62)
	P5–9	Barrel cortex	CX3CR1	Hoshiko et al. (11)
	P5	dLGN of thalamus	C3, activity dependent	Schafer et al. (36)
	P2	POA	Sex difference, PGE2, sex behavior	Lenz et al. (33)
	P30+	Motor cortex	BDNF, motor learning	Parkhurst et al. (52)
	P40	dLGN of thalamus	Second wave of synaptic pruning	Schafer et al. (48)
	P15	Spinal cord and thalamus	IL-33m, sensorimotor behavior	Vainchtein et al. (47)

Cell genesis	P2–5	Ventricular area	IL6, IL1B, TNF, IFNY	Shigemoto-Mogami et al. (37)

Myelinogenesis	P6–22	Corpus callosum, cerebellum	IGF1, Cd11c+	Hagemeyer et al. (32)
	P6–22	Corpus callosum	IGF1, Cd11c+	Wlodarczyk et al. (26)

Cellular phagocytosis	E9.5	Neural tube	Earliest noted microglia function	Kierdorf et al. (4)
	P0-2	Hippocampus	Induce cell death, Cd11b	Wakselman et al. (40)
	E17–P6	Cortical proliferative zones	Phagocytosis of progenitor cells	Cunningham et al. (31)
	P4	Hippocampus	VEGFR1, Nox-2	Lelli et al. (41)
	P2–3	Hippocampus	Sex difference	Nelson et al. (28)

Axon dynamics	E14.5	Striatum	Cr3, Dap12/Tyrobp, Cx3cr1	Squarzoni et al. (38)
	E17.5	Corpus callosum	Dap12	Pont-Lezica et al. (35)

Cell positioning	E18.5	Cortex		Squarzoni et al. (38)

Cell survival	P3–4	Cortex	IGF1, Cx3cr1	Ueno et al. (39)

Despite this knowledge, we have only scratched the surface of understanding microglia function during development, and the situation is likely more complicated than it seems. Microglia support cell survival in layer V of the developing neocortex and yet oligodendrocytes do so as well (39, 53). Thus, microglia could be contributing to cell survival indirectly by supporting oligodendrogenesis or through an additive effect on oligodendrocyte-derived growth factors. Microglia also support cell genesis in the developing brain through release of several cytokines (37), and it is unknown whether cytokine receptors are expressed on neural progenitor cells during development. These receptors would be necessary for direct stimulatory effects of cytokines on neurogenesis to occur. These studies highlight the many microglia functions during brain development and the challenge of clearly determining which microglia function is important to a given developmental process in the brain. Additionally, the implications for lifelong brain function and behavior remain unknown in many of these cases.

## Microglia and Psychiatric Disorders of Development: Synaptic Pruning and Beyond

As introduced above, microglia are posited to regulate normal and abnormal brain development seen in neuropsychiatric disorders primarily *via* the regulation of synaptic pruning. The strongest evidence that microglia regulate developmental synaptic pruning is the aforementioned studies of the complement cascade in the thalamus. Recently, it was also found that specific complement component 4 (C4) alleles are highly associated with schizophrenia and that C4 knockout in mice resulted in decreased synaptic pruning in the visual thalamus (54). These data support the notion that dysfunction within the complement system may perturb microglia-mediated synaptic pruning and contribute to the onset of neurodevelopmental disorders such as schizophrenia.

An exciting remaining question is how C4 risk variants, or other complement system manipulations, shape behavior relevant to psychiatric disorders in rodents or humans. Recent evidence indicates that C4a is upregulated in the post-mortem tissue of autistic and schizophrenic patients (55). However, C1q and C3 knockout mice only begin to show changes on some tasks when they are aged, although C1q knockout mice do display increased connectivity (56–58). For other regulators of the complement pathway, such as CUB and Sushi Multiple Domains 1 (CSMD1), an inhibitor of the complement cascade, some studies show they contribute to behavior changes relevant to schizophrenia, whereas others do not (59–61). Recent research indicates that synaptic pruning deficits can alter behavior. IL-33 drives microglia synaptic pruning in the spine and the thalamus, and IL-33 knockout results in altered sensorimotor behavior, but not motor or auditory function (47). The strong association of specific C4 alleles with schizophrenia presents an interesting and unique opportunity to directly connect microglia synaptic pruning with the development of schizophrenia. For example, do specific C4 alleles segregate with specific behavioral/functional impairments (endophenotype/biotype) in humans diagnosed with schizophrenia or another psychotic disorder? If so, microglia synaptic pruning mediated by the complement cascade could be directly connected to specific behavioral/functional impairments in humans.

The data to support the possibility that disruptions in normal microglia-mediated synaptic pruning could contribute to neuropsychiatric disorders are strong so far, yet our previous discussion on the diverse aspects of brain development shaped by microglia is worth keeping in mind. The question that needs to be answered is which of these functions is important for microglia-mediated programming of behavior. Several lines of evidence suggest that we need to be cautious about prematurely concluding that microglia primarily shape brain development and possibly mental health disorders primarily *via* underlying effects on synaptic pruning. For example, disrupting fractalkine signaling during development has been linked to deficits in social behavior that may reflect an autistic-like phenotype (62), and these deficits are posited to occur through dysregulated sculpting of neural circuits (12). Interestingly, however, fractalkine signaling also regulates cortical oligodendrogenesis (63). White matter irregularities are common in people diagnosed with a mental health disorder, including autism and schizophrenia (64–68). Microglia also control axonal fasciculation, which may also contribute to functional connectivity differences between brain regions, including those seen in CX3CR1 knockout mice (35, 38, 62). Microglia are also necessary for spine synapse formation and synapse maturation during development, and this support of spinogenesis and synapse maturation is important for behavior (11, 33, 34, 52). Other cells also contribute to synaptic pruning in the developing brain. Astroglia phagocytize synapses in the dLGN to a greater extent than do microglia (69).

Moving forward, we must consider the behavioral implications of microglia manipulations and attempt to tease out the specific mechanisms through which microglia are influencing behavioral development. With the discovery of microglial-specific genes that are distinct from systemic-macrophage genes, we can begin to use genetic tools to specifically target and manipulate microglia. In this way, we will begin to directly test how microglia influence the development of brain and behavior, whether they do so through synaptic pruning, regulation of myelination or support of neurogenesis, or (as is likely) a combination of all these routes.

## The Microenvironment Drives Microglia Function: Heterogeneity Abounds

Microglia in the brain are not interchangeable. Peripherally, macrophage function in different tissues is driven by the tissue microenvironment (70, 71). In the adult brain, the microenvironment drives the microglia phenotype in both rodents and humans (72–74). Many factors, such as CSF1, IL-34, TGF-β, cholesterol, CX3CL1, PGE2, and VEGF, are released in discrete brain regions to drive specific microglia functions during development (8, 10, 33, 36, 41, 75). Several studies have shown that TGF-β and CSF1-R signaling are important for microglia development, survival, and identity (6, 72, 76, 77). VEGF, potentially released by neural progenitors, can recruit CD68 + phagocytic microglia during development or induce a phagocytic phenotype (41, 75). Astrocytes seem to be an important mediator of microglia function as well, with astrocyte-conditioned medium supporting microglia survival *in vitro* (72). Interestingly, microglia gene expression is dramatically altered by removing microglia from the brain and culturing them. Culturing microglia increases both inflammatory and developmental genes even in the presence of important astrocyte-derived survival factors (TGF-β, CSF1, and cholesterol) (72). Conversely, cultured microglia rapidly adopt a normal *in vivo* gene expression profile when transplanted into the adult brain (72). Turano et al. (78) showed that microglia inflammatory gene expression in response to immune challenge *in vitro* was different depending on whether other neural cells were also present in culture, suggesting these other cells regulate microglia behavior. There are several neurotransmitters that locally regulate microglia in the adult brain such as nucleotides (ATP, UDP, etc) and histamine (79–81). However, it is unknown how neurotransmitters regulate microglia function during development. These results drive the important point that multiple, locally derived factors regulate microglia identity and function.

The trajectory and specific signaling mechanisms governing brain development are region-dependent (82, 83). However, very little is known about regional differences in microglia during development. We have found that microglia density in the amygdala is higher than it is in the medial prefrontal cortex during the first week of life (84). In contrast, sub-regional differences in microglia function, density and morphology in the basal ganglia do not occur until after 2 weeks of age (73). Similarly, there are regional differences in the timing of Tmem119 expression in the brain, which appears to be a good proxy marker of an adult microglia phenotype (14), suggesting that microglia mature at different rates across the brain. These differences in microglia density and gene expression might reflect the different developmental trajectories of specific brain regions. In addition, as will be discussed further below (see Microglia and Sex Differences: Ignored Phenotypic Differences With Big Implications), there are also prominent sex differences in microglia number, function, and phenotype during brain development. Thus, hormonal signals could also differentially affect the microenvironment during development, depending on regional differences in hormone receptor expression. Together, these results indicate that there are other uncharacterized CNS intrinsic cues that direct microglia phenotype during development and across tissues.

Alterations in microglia function that result from upstream changes in the CNS environment may also be relevant to understanding the pathophysiology of human psychiatric disorders. Post-mortem brain tissue from individuals with autism is enriched with many gene variants or mutations that are associated with synaptic and neuronal genes, but not microglia-related genes (85–87). However, many genes with altered expression in the same tissue are expressed by microglia and astroglia (85–87). These data suggest that microglia respond to an altered neural environment induced by non-glial risk variants. But the verdict is still out: Recently, conflicting evidence in rodent models of Rett Syndrome studies has arisen. Some evidence suggests that direct microglia dysfunction is implicated in pathology seen in a mouse model of Rett Syndrome (88); other evidence suggests that microglia are downstream responders to an altered brain microenvironment (48, 89). In many cases, it will likely turn out to be not “either/or” but “both”— in other words, microglia may directly drive neuropathology as well as respond to an altered brain microenvironment. The ability of microglia to adapt to the brain environment suggests that microglia function can be built-to-suit, thus generalizations between microglia during development, normal adulthood, or various pathological conditions may be hard to come by.

## The Macro-Environment Drives Microglia Function

As innate immune cells, microglia are highly responsive to environmental perturbations. We define the macro-environment as large-scale perturbations such as stress or immune challenge that change the microenvironment of the body and brain specifically. Early life perturbations are major risk factors for many psychiatric and neurological disorders, suggesting that brain development is altered by these experiences (90). And yet, there has been very little work that specifically assesses microglia function acutely in the hours, days, and weeks immediately following a macro-environmental perturbation. Most research to date has instead focused on enduring changes in microglia of adults following an early life insult. What research has been performed on acute effects of early life perturbations on microglia has been very interesting. Prenatal or early life immune activation accelerates the maturation of microglia rather than inducing a “pro-inflammatory” phenotype (24, 91). There may also be differences in how microglia function is changed depending on whether immune challenge is initiated using a viral mimetic challenge, such as Poly I:C or a bacterial endotoxin challenge with LPS. Maternal immune challenge with Poly I:C does not seem to induce as much of a “pro-inflammatory” phenotype in microglia compared to LPS (31, 92). However, several studies have found that prenatal Poly I:C and neonatal LPS both accelerate the maturation of microglia (24, 91). Perturbations of the gut microbiome during development can lead to underdeveloped microglia (24, 93, 94). Males and females may also show differential responses to developmental perturbations. For example, male microglia show more dramatic changes in gene networks compared to females when developing in germ-free conditions or after neonatal endotoxin challenge (91, 94). Others have found that prenatal maternal diesel exhaust exposure changes microglia-neuron positioning in the offspring brain, similar to that seen in autism (95, 96). Early life stress is associated with many prominent microglia changes, including downregulation of genes normally expressed in immature microglia, a temporary increase in microglia density in the immature brain, and increased phagocytic activity (29).

Given the heterogeneity in the microglial response to different early life perturbations or timing in these perturbations, it is critical to consider whether the crucial factor that contributes to brain development is altered microglia number (e.g., microglial load), altered microglia function, or both. While many studies have focused on microglia function during development, changes in microglia density could also drive behavioral changes. We and others have found that temporarily depleting microglia during the early neonatal period programmed long-term changes in behavior, including decreased anxiety-like and despair-like behavior, and working memory deficits (84, 97). CX3CR1^−/−^ mice have a transiently decreased microglia density in the hippocampus and delayed microglia entry into the barrel cortices and social behavior (11, 12, 62). In contrast, microglial loss in adulthood seems to have little impact on behavioral outcomes (77). It is important to note that the changes in microglia density are temporary and need not be permanent to have lasting effects. Changes in microglia density could mean that there is a change in the magnitude of microglia’s influence on brain development (e.g., fewer microglia, less synaptic pruning), without overt changes in microglia function. Conversely, changes in microglia density could be compensated for by changes in microglia function (e.g., more microglia, less synaptic pruning per microglia) or vice versa. Changes in density due to prenatal/early life challenges could also reflect changes in maturation of microglia. We must begin to determine the functional and behavioral changes that occur following specific targeted microglia manipulations to significantly advance the translational impact of the field of developmental neuroimmunology.

## Microglia and Sex Differences: Ignored Phenotypic Differences with Big Implications

During ontogeny, the brain is permanently organized as male- or female-typical in a process called sexual differentiation. Sex-specific brain development supports the emergence of behaviors necessary for reproduction, parenting, and social behaviors such as aggression [reviewed in Ref. (98)]. Sexual differentiation is driven by sex-specific hormonal signals. In mammals, the major hormonal signal is the androgen testosterone, which is secreted by the fetal testes (99). In human males, testosterone secretion occurs prenatally, and this testosterone enters the developing brain and binds to androgen receptors, which act as transcription factors to shape sex differences in gene expression. In humans, the process of sexual differentiation is largely completed by birth. In rodents, the major elements of sexual differentiation are the same, with several notable exceptions. Unlike in humans, the rodent testis begins secreting testosterone on E18, 3 days prior to birth, and testosterone secretion ends during the first postnatal day (100). However, the critical period in which the brain remains sensitive to the early programming effects of hormones extends until approximately P10. Also unlike humans, in rodents, testosterone is converted into another steroid hormone, 17-beta estradiol, in the brain by the enzyme p450 aromatase, and estradiol binds to estrogen receptors in the brain to effect male-typical brain organization (101, 102). In females (both human and rodent), the process of sexual differentiation proceeds along a “default path” in the absence of an active hormonal signal (103), but also has a similar critical period.

Many of the major processes of brain development proceed differently in males and females, including cell genesis, cell death, cell migration, axon guidance, synaptic patterning, and myelination (see McCarthy et al. (104) for thorough review). Sex differences in both microglia number and their properties have been documented in the developing brain. We have thoroughly reviewed these sex differences in microglia and inflammatory mediators elsewhere, both in the context of normal brain development as well as in response to early life perturbations [see Nelson and Lenz (105).]. Nevertheless, several important points are worth repeating, especially given the recent National Institutes of Health mandate that all studies consider the contribution of biological sex to their results.

In many brain regions, males have more microglia in the developing brain and tend toward a more ameboid morphology (25, 33). This may indicate either greater activation of microglia in males or possibly a more immature phenotype (25, 33, 91). Indeed, female microglia appear to mature and reach an adult phenotype earlier in development than do male microglia (91). There are also sex differences in microglial phagocytosis in the rat hippocampus, with females having higher levels of phagocytosis and phagocytic gene expression than males (28). In this case, female microglia engulf neural progenitor cells at higher rates than in males (28), suggesting that microglia could regulate a known sex difference in neurogenesis in the developing hippocampus (106). Sex differences in microglia are not present in the rodent brain prior to the onset of the testicular androgen surge, but are seen soon after the surge occurs (25). Treating females with male-typical hormones (estradiol or testosterone) during the critical period for sexual differentiation induces a male microglia phenotype within days, indicating microglia are responding to steroid hormones (28, 33). However, in both our work and that of other research groups, steroid hormone receptor expression is either extremely low or undetectable in microglia in the developing brain (33, 78, 107). This suggests that crosstalk between microglia and other steroid-sensitive cells is necessary for microglia sexual differentiation to occur. It remains to be determined how the sex differences in microglia number are programmed. Differential chemotactic signals attracting microglia to particular brain regions are one possibility (25). Another possibility is differential proliferation or cell death, yet we have not seen sex differences or hormonal regulation of microglia proliferation in the developing hippocampus (28).

Not only are microglia targets of the sexual differentiation process, but they are also key effectors of sexual differentiation. Many of the mechanisms through which sexual differentiation occurs (e.g., cell genesis, cell death, synaptic patterning, and myelination) are regulated by microglia. Thus, a natural question is whether microglia contribute to the sexual differentiation process. Although this question has only been addressed in select few studies, it does appear to be the case. In the rodent preoptic area (POA), there is a sex difference in synaptic patterning that is organized by exposure to neonatal androgens, such that neurons in the male POA have two to three times the number of dendritic spine synapses than do neurons in the female POA (108, 109). A higher level of dendritic spines in the male POA persists until adulthood, and the number of dendritic spines correlates positively with the number of male-typical mounting behaviors in adult mating tests (109, 110). This developmental sex difference depends upon estradiol, as well as its downstream effector, the inflammatory molecule prostaglandin E2 (PGE2). If female rodents are treated with either estradiol or PGE2, they will show male-typical dendritic spine density in the POA as well as male-typical mounting behavior in adulthood (109). Microglia are necessary players in this process in the POA. Estradiol-induced masculinization of PGE2 levels, dendritic spines as well as male-typical adult sexual behavior can be blocked by concurrent administration of the microglial inhibitor, minocycline, during development (33). Temporary ablation of microglia from the developing brain leads to diminished performance of males, though not females, on sexual behavior tests in adulthood (97).

Another study of the nearby anteroventral periventricular nucleus (AVPV) of the POA also suggests immune regulation of sexual differentiation. The AVPV is smaller in males due to hormonally induced acceleration of cell death in the developing male, and in females, the nucleus is responsible for adult hormonal cycling (111). In the AVPV, a sex difference in the immune factor TNF family member repressor protein (TRIP) drives this sex difference (112). The cellular source of this immune factor has not been determined, but may well be microglia. The previously described sex differences in microglia properties in the immature brain suggest that many more such instances are yet to be uncovered in which microglia contribute directly to sexual differentiation of brain and behavior.

In rodents, diverse challenges such as early life bacterial infection (113), prenatal high fat diet (114), intrauterine inflammation (115), early life stress (116–118), and prenatal exposure to diesel particulate (95) all elicit either microgliosis or increased microglia number in the developing offspring brain. In response to these varied early life perturbations, male rodents tend toward greater microglial reactivity in the brain as well as greater inflammatory gene expression than females. In addition, transcriptome profiling shows that challenge with LPS accelerates microglial maturation index in males, but not females, again suggesting that males are more vulnerable to inflammatory insults during this period (91). This same study used transcriptome data analysis of human brain samples and found that the microglial developmental index was accelerated in postmortem tissue from individuals with autism spectrum disorder (ASD) and Alzheimer’s, indicating that this sex-specific acceleration of microglia maturation may be relevant to sex differences in human disease pathophysiology. Microglia from germ-free mice also show a sex difference during development and in adulthood (94). Male microglia show more gene expression changes during development whereas female microglia show more gene expression changes during adulthood in response to a loss of intestinal microbes. Given that two different developmental perturbations resulted in more changes in male microglia, it will be interesting to see whether this same sex difference in microglia is generalizable to other developmental perturbations.

In the context of human neurodevelopmental disorders, sex differences in microglia or other immunocompetent cells may be central. Many brain-based disorders of development, including autism, attention deficit hyperactivity disorder (ADHD), Tourette disorder, and schizophrenia [reviewed in Ref. (104)]. Autism is one of the most sex-biased disorders, though the reasons for this sex bias are unknown. Early life inflammatory experiences increase the risk for neurodevelopmental disorders (119–123), thus immune mediators may contribute to this sex bias. A study of postmortem brain tissue from autistic individuals has shown that there are sex differences in astrocyte and microglia markers in post-mortem autistic brain, but not in autism risk genes (124). This means that sex differences in autism risk gene expression are not likely responsible for the higher rate of autism in males. Instead, it suggests that microglial and astrocytic genes are more highly expressed in the male brain independent of autism risk genes to increase risk for autism and possibly other comorbid disorders of brain development. However, others have found that, while isolated murine microglia show a sex difference during development, isolated human microglia do not (94). Further research is needed to determine whether microglia contribute to the sex differences in human neurodevelopmental disorders.

## What about Microglia in Humans?

A majority of research on microglia has been performed in rodent models, and to date, relatively little research has been done to determine whether microglia function is similar in humans. Several commentaries have addressed the differences between murine and human microglia and the immune system [see reviews (125–127)]. Here, we highlight the important differences and similarities in microglia function across species during development as well as analysis of microglia in neurodevelopmental disorders. Only 30% of microglia genes in humans are enriched in mouse microglia (128); however, there is a core set of important microglia genes whose expression is conserved between humans and mice (94). There are several differences in molecules that regulate phagocytosis such C4a, C4b, and Siglec-11 which are present in humans, but not mice (mice do express a single C4 isoform), and Siglec-H and Fcrls which are present in mice, but not humans (54, 76, 129, 130). Studying the functional impact of genes that are either differentially expressed or not expressed at all in one or the other species will likely prove challenging, especially when these differences are highly associated with neurodevelopmental disorders. However, there are some crucial similarities that justify continued use of rodent models to study microglia function during development. Microglia colonize the human brain over a similar timeline to that in rodents (2, 131). Microglia phagocytize progenitors in mice and primates in the ventricular areas and exhibit similar increases in phagocytic microglia as the progenitor proliferation begins to decrease (31). We also know that microglia function is similarly shaped by the microenvironment, even if the microenvironment is slightly different between humans and rodents (19, 24, 74). With the development of new and better tools, we can likely identify where the crucial differences are and how to account for them experimentally.

Neuroinflammation in human neurological or neuropsychiatric disorders has been assessed using PET scanning with a ligand to visualize the translocator protein (TSPO) on microglia in the brain (132). Such studies have shown increases in putative microglia activation in many neuropsychiatric disorders, including autism and major depression (133, 134). These studies certainly indicate a change in glia, but alone do not indicate how microglia function is altered in these conditions (132). For example, TSPO signal has been seen to decrease in schizophrenia despite other evidence of neuroinflammation in the condition (135). In addition to microglia, TSPO is expressed in activated astrocytes, and TSPO expression is not solely affected by inflammation, but rather by the specific microenvironment (135). Since these differences in TSPO binding in neuropsychiatric disorders likely mean some change in glial function has taken place, future research should focus on more in-depth analysis to assess the specific functional changes in microglia (or astrocytes) in these disorders.

We also know very little about what microglia are doing during development in humans who go on to develop a psychiatric disorder later in life. Part of the challenge is that many changes in microglia function and related brain development are likely to occur well before symptoms are present. Several postmortem studies have found increased number and reactivity of microglia in developmental disorders, including autism, schizophrenia, and Tourette disorder (136–139). Transcriptome analysis of post-mortem tissue from individuals with autism and Tourette disorder shows that the differentially expressed genes are highly enriched in microglia and/or immune genes (124, 138). It should be noted that not all studies of post-mortem tissue have found dramatic changes in microglia density or morphology (137, 139). Additionally, it has not been determined whether microglia changes observed within the postmortem brains of individuals with neurodevelopmental disorders are part of the etiology and pathophysiology of the disorder or instead a downstream response to a dysfunctional or deteriorating brain environment.

Despite these caveats, several pieces of data converge to make a strong case that microglia dysfunction is actively contributing to the pathophysiology of neurodevelopmental disorders. First, the previously discussed schizophrenia study implicating a complement system risk variant in the disorder (54); second, the Rett syndrome study showing that microglia manipulations are therapeutically effective in the rodent model of the disorder; and third, the autism study showing higher expression of glial genes in males with autism (124). Fourth, a very recently published paper shows substantial overlap in neuronal gene modules associated with several neuropsychiatric disorders, but that autism is associated with a unique upregulation in a glial-related gene expression module (55). What is more, the microglia modulate upregulated in ASD includes IRF8, a transcription factor important for the transition from pre-microglia to early microglia, which suggests that the development of microglia may be altered in ASD (4, 55).

## Other Immunocompetent Cells in the Brain: In Conversation with Microglia?

Microglia are by far the most abundant immune-derived cell type in the brain. Yet other innate immune cells have also been detected within the healthy developing brain. Of note are mast cells, which are tissue-resident innate immune cells that are similar to basophils. Mast cells have been detected in the rodent and human brain under healthy conditions. Interestingly, in the healthy human brain, mast cells are most often detected within the developing brain (140) but their function is largely unknown. In mice, mast cells are detectable throughout the lifespan (141). Mast cell-deficient mice show several abnormalities that suggest their function is crucial in the developing or adult brain. For example, mast cell deficient mice show deficits in learning and memory, hippocampal neurogenesis, and increased anxiety-like behavior in adulthood (141, 142). Mast cells are a potent source of amine neurotransmitters such as serotonin and histamine (143) and may function as neuromodulatory cells. However, mast cells also release a host of inflammatory molecules, including cytokines, chemokines, and prostaglandins (143), all of which can regulate brain function in healthy or inflammatory conditions. Mast cells may also regulate microglial activation *via* secretion of these mediators, with recent *in vitro* studies showing that conditioned mast cell medium can induce the release of pro-inflammatory cytokines in cultured microglia (144). Antagonizing histamine receptors, proteinase activated receptors, and toll like receptor (TLR) 4 prevented these effects on microglia, suggesting that multiple mast cell mediators may be involved in mast cell-microglia crosstalk. Future studies are necessary to determine how crosstalk between different immune cell types occurs *in vivo* and whether this crosstalk is important for normal brain development or abnormal development following early life perturbations.

Astrocytes also make up a huge proportion of cells within the brain. Although derived from neural stem cells in neurogenic niches in the brain (145), astrocytes are immunocompetent insofar as they release and respond to immune system mediators (such as cytokines) and are capable of antigen presentation [reviewed in Dong and Benveniste (146)]. But what is the nature of crosstalk between microglia and astrocytes? In neurodegenerative studies, crosstalk between the two cell types has been well demonstrated, often with activated microglia inducing a neurotoxic phenotype in astrocytes (147). Environmental perturbations that lead to altered microglial gene expression and microgliosis in the brain, such as immune challenge with LPS, also induce the release of proinflammatory mediators from astrocytes (148, 149). Interestingly, astrocytes also prune synapses during development, suggesting that microglia could indirectly change synaptic pruning by releasing cytokines or other signals that alter astrocyte function (69, 147). What has been largely lacking to date in developmental neurobiology research is any careful investigation of chicken-and-egg relationships, including specific signals between microglia and astrocytes that regulate their function in either direction. A very recently published article, however, has taken an important step in this direction, showing that astrocyte-derived IL-33 drives microglia phagocytic activity in the developing central nervous system (47).

While the healthy brain does not contain large quantities of peripherally derived immune cells, recent evidence nevertheless suggests that peripheral cells can influence normal brain function. One potential route is *via* effects in the meninges that are conferred across the blood brain barrier. Adaptive immune cells, such as T cells, are present within the meninges, and they regulate brain function and the display of social behavior *via* interferon signaling across the blood brain barrier (150). T cells have also been implicated in brain and behavioral development, particularly in sexual differentiation. T cell-deficient mice display altered size of several areas of the brain, including the hypothalamus, amygdala, periaqueductal gray, and raphe nuclei (151). Additionally, T cell-deficient animals have a loss of sexual dimorphism in the size of several brain regions, such as the bed nucleus of stria terminalis, with females resembling wild-type males (151). Finally, these animals show decreased anxiety, suggesting that T cells are necessary for programming of mood related behavior. The mechanisms through which T cells influence brain and behavioral development are yet to be determined, though it may well be that T cell-derived signaling across the blood brain barrier influences microglia function during the critical period for brain organization.

## Conclusion and Future Questions

As with any young field, it is important not to conclude too early that we truly understand how microglia shape brain development. What we do know is that microglia make up a significant percentage of cells in the brain throughout life and that they are as important in healthy conditions as they are in pathological conditions. We have attempted to move beyond summarizing the role microglia are known to play in brain development and function in order to point out that in many cases, we know very little and much more basic discovery work is needed. We highlight the diversity of microglia function in the developing brain to emphasize their potential importance to understanding and treating brain-based disorders of development. At this stage, we have few sophisticated tools to manipulate microglia function beyond cell-type specific knockout models. Thus a future goal of the field should be to develop new tools to manipulate microglia function, both phagocytic function and release of secreted factors, in targeted ways to connect microglia behavior to structural, functional, and behavioral outcomes in living organisms. It is an exciting time to be researching neuroimmune function, and the promise that microglia could be viable targets to prevent or treat brain based disorders is high.

## Author Contributions

KL and LN planned and wrote the manuscript.

## Conflict of Interest Statement

The authors declare that the research was conducted in the absence of any commercial or financial relationships that could be construed as a potential conflict of interest.
